# Altered Blood Brain Barrier Permeability and Oxidative Stress in *Cntnap2* Knockout Rat Model

**DOI:** 10.3390/jcm11102725

**Published:** 2022-05-11

**Authors:** Idil Memis, Rahul Mittal, Emily Furar, Isaiah White, Rebecca S. Eshraghi, Jeenu Mittal, Adrien A. Eshraghi

**Affiliations:** 1 Hearing Research and Communication Disorders Laboratory, Department of Otolaryngology, Neurotology Division, University of Miami Miller School of Medicine, Miami, FL 33136, USA; idil_memis@hotmail.com (I.M.); r.mittal11@med.miami.edu (R.M.); emilyfurar@mail.usf.edu (E.F.); ixw91@miami.edu (I.W.); rebeccaeshraghi@gmail.com (R.S.E.); j.mittal@med.miami.edu (J.M.); 2 Department of Neurological Surgery, University of Miami Miller School of Medicine, Miami, FL 33136, USA; 3 Department of Biomedical Engineering, University of Miami, Coral Gables, FL 33146, USA; 4 Department of Pediatrics, University of Miami Miller School of Medicine, Miami, FL 33136, USA

**Keywords:** autism spectrum disorder, *Cntnap2*^−*/*−^ rats, oxidative stress, blood brain barrier, animal model, tight junction proteins, nitric oxide

## Abstract

Autism spectrum disorder (ASD) is a neurodevelopmental disorder characterized by three core symptoms, specifically impaired social behavior, stereotypic/repetitive behaviors, and sensory/communication deficits. Although the exact pathophysiology of ASD is still unknown, host genetics, oxidative stress, and compromised blood brain barrier (BBB) have been implicated in predisposition to ASD. With regards to genetics, mutations in the genes such as *CNTNAP2* have been associated with increased susceptibility of developing ASD. Although some studies observed conflicting results suggesting no association of *CNTNAP2* with ASD, other investigations correlated this gene with autism. In addition, CNTNAP2 mediated signaling is generally considered to play a role in neurological disorders due to its critical role in neurodevelopment, neurotransmission, and synaptic plasticity. In this investigation, we studied BBB integrity and oxidative stress in *Cntnap2*^−*/*−^ rats. We observed that the BBB permeability was significantly increased in *Cntnap2*^−*/*−^ rats compared to littermate wild-type (WT) animals as determined by FITC-dextran and Evans blue assay. High levels of thiobarbituric acid reactive substances and lower amounts of reduced glutathione were observed in brain homogenates of *Cntnap2*^−*/*−^ rats, suggesting oxidative stress. Brain sections from *Cntnap2*^−*/*−^ rats showed intense inducible nitric oxide synthase immunostaining, which was undetectable in WT animals. Quantification of nitric oxide in brain homogenates revealed significantly high levels in *Cntnap2*^−*/*−^ rats compared to the control group. As increased permeability of the BBB and oxidative stress have been observed in ASD individuals, our results suggest that *Cntnap2*^−*/*−^ rats have a high construct and face validity and can be explored to develop effective therapeutic modalities.

## 1. Introduction

Autism spectrum disorder (ASD) is a heterogenous group of complex neurodevelopmental disorders that begin early in childhood [1,2,3]. According to the US Centers of Disease Control and Prevention (CDC), the incidence of ASD is about 1 in 44 children and is steadily increasing [4]. The core features of ASD include persistent deficits in social communication and interaction as well as restricted, repetitive patterns of sensory-motor behavior, interests, or activities accompanied with speech-language delay [2,5]. Despite advances in the medical field, there are still no effective treatments available for ASD, which can be attributed to the incomplete understanding about the pathophysiology of this neurological disorder. A wide arsenal of factors such as epigenetic changes, immune system dysfunction, hormone imbalance, environmental toxins, gastrointestinal factors, and disturbance of brain energy metabolism are considered to contribute to the etiology of ASD [6,7,8,9,10].

The advent of transgenic technologies has revolutionized the field of ASD by providing genetic mouse models to understand the pathogenesis of neurological disorders including ASD [11,12]. Although mouse genetic models have been invaluable for studying underlying neurobiological mechanisms, mouse behavioral analyses have often been challenging. Rat models are more appropriate than mice for understanding ASD pathophysiology as they exhibit complex social behavior [13]. Normal young rats are playfully aggressive creatures, wrestling, boxing, and pinning their siblings down by the neck unlike mice. In addition, the metabolic physiology of rats is more closer to humans unlike in mice. Furthermore, rats have a larger brain volume to perform neurobiochemical, electrophysiological, and neuroanatomical studies This makes the rat a more suitable animal to understand the molecular processes involved in ASD etiology. 

Besides a number of predisposing factors as mentioned before, genetics has been strongly implicated in the pathophysiology of ASD. In this regard, mutations in genes such as contactin associated protein like 2 gene (*CNTNAP2*) have been associated with ASD [14,15,16,17,18,19,20,21,22,23]. *CNTNAP2*, which codes for the neurexin CASPR2, plays a crucial role in the development of the nervous system, in synaptic functions, and neurotransmission [24,25,26]. Although studies have suggested association or predisposition to ASD in individuals with *CNTNAP2* mutations [14,15,16,17,18,19,20,21,22,23], the role of *CNTNAP2* in the pathophysiology of autism is still not clear. Studies have reported conflicting results regarding the association of *CNTNAP2* with ASD [27,28,29]. A study suggested a limited or likely neutral role of *CNTNAP2* in the susceptibility to develop psychiatric disorders including ASD using a large database set including 4,483 ASD subjects and 13,042 controls [27]. A family-based association study suggested that *CNTNAP2* polymorphisms might not be associated with autism [28]. Another study suggested that there is no evidence for statistically significant association of rare heterozygous mutations in any of the *CNTN* or *CNTNAP* genes, including *CNTNAP2* with ASD [29]. Considering conflicting results, further studies are needed to decipher the precise contribution of *CNTNAP2* in ASD. 

In addition to host genetics, oxidative stress is another important player in the etiology of ASD [8,30,31,32,33,34,35]. The imbalance between antioxidant defenses and the production of reactive oxygen species (ROS) as well as reactive nitrogen species (RNS) lead to oxidative stress, which can cause damage to cellular and neuronal structures in the brain [36]. ROS and RNS are products of cellular metabolism and primarily, they include superoxide radical (O^2−^), nitric oxide radical, hydroxyl radical, hydrogen peroxide (H_2_O_2_), and peroxynitrite radicals [36,37]. Postmortem brain tissues, urine, plasma, and serum samples from ASD individuals show markers for the production of ROS and RNS as well as impaired glutathione activity, which provides protection against oxidative stress [33,38,39,40,41,42,43]. In the genetically modified mouse models, which have a similar oxidant-antioxidant profile to individuals with ASD, it is also shown that a decrease in the glutathione causes an increase in repetitive autism-like behaviors [44]. 

Nitric oxide (NO) is one of the most important neurotransmitters in both the central nervous system (CNS) and the peripheral nervous systems (PNS). It is synthesized from L-arginine via nitric oxide synthase (NOS) in the presence of NADPH and oxygen. Three isoforms of NOS have been identified, namely neuronal NOS (nNOS), endothelial NOS (eNOS), and inducible NOS (iNOS) [45]. Under physiological conditions, NO levels are regulated by two of these NOS isoforms, nNOS and eNOS. nNOS is constitutively expressed in the cytosol of neurons, and eNOS is expressed in endothelial cells in the membrane [46,47]. Under pathological conditions such as infection, trauma, or ischemic insult, iNOS can be expressed in the brain and regulate the level of NO [45,48,49]. Elevated NO production has been observed in urine, saliva, plasma, and serum samples from individuals with ASD [50,51]. 

The brain is protected from the detrimental effects of ROS and RNS due to the presence of the blood brain barrier (BBB). The BBB is a continuous endothelial membrane which maintains CNS homeostasis by tightly controlling the passage of oxygen, carbon dioxide, nutrients, and ions [52,53,54,55,56,57]. The disruption of the BBB is a hallmark of ASD [58]. The BBB disruption allows immune components, neurotoxic debris, cells, and pathogens to access the brain. Studies have reported increased levels of pro-inflammatory cytokines such as tumor necrosis factor alpha (TNF-α), interleukin-6 (IL-6), interleukin-8 (IL-8), and macrophage chemoattractant protein-1 (MCP-1), accompanied with activation of microglia and astroglia in the postmortem brain tissue from ASD individuals [59,60,61]. These might potentially contribute to the clinical manifestations of ASD by hampering the normal function of the brain. 

Studies have shown that deletion of *Cntnap2* in animal models leads to behavioral, cognitive, neuronal, and sensory alterations similar to those seen in individuals with ASD [62,63,64,65,66]. The behavior profile of *Cntnap2*^−*/*−^ rats has already been extensively characterized in previous studies and it has been shown that these rats exhibit an ASD-like phenotype [66]. *Cntnap2*^−*/*−^ rats have been shown to demonstrate deficits in sociability and social novelty using a three-chamber behavior test. Enhanced acoustic startle responses, greater aversions to sound at moderate intensity and lack of rapid audiovisual temporal recalibration has been observed in *Cntnap2*^−/−^ rats. These findings suggest sensory processing deficits in *Cntnap2*^−*/*−^ rats at both the pre-attentive and perceptual levels similar to the phenotype observed in individuals with ASD. In addition, altered auditory processing and behavioral reactivity have been observed in *Cntnap2*^−*/*−^ rats accompanied with deficits in sensorimotor gating. However, the molecular mechanisms such as oxidative stress that can lead to ASD-like phenotype have not been characterized in previous investigations. The objective of the present study was to determine whether *Cntnap2* deletion affects the BBB permeability as well as determining if it leads to oxidative and nitrosative stress using a rat model. 

## 2. Materials and Methods

### 2.1. Animals

Heterozygous breeders of *Cntnap2*^−*/*−^ rats on Sprague Dawley background were obtained from the Envigo company (Indianapolis, IN, USA). The model contains a five base pair deletion in exon six of the Cntnap2 gene, created using the zinc finger nuclease target site CAGCATTTCCGCACC|aatgga|GAGTTTGACTACCTG. All experimental animals were obtained from heterozygous crossings. Both male and female rats were used in each experiment. The study protocol was approved by the Animal Care and Use Committee of the University of Miami and is in full compliance with the NIH guidelines for the care and use of laboratory animals.

### 2.2. Fluorescein Isothiocyanate (FITC)-Dextran Assay 

To determine the integrity of the BBB, we performed the FITC-dextran assay [67]. FITC-dextran (4 kDa; 500 mg/kg, Sigma, St. Louis, MO, USA) was administered intraperitoneally to the rats. The rat brains were then harvested 6 h post-administration after transcardial perfusion with PBS (Sigma, St. Louis, MO, USA) and fixed in 4% paraformaldehyde (Electron Microscopy Sciences, Hatfield, PA, USA) at 4 °C for 24 h. The brains were sectioned and subjected to confocal microscopy to determine FITC-dextran extravasation. Five fields per section and three sections per animal were analyzed. To determine the mean signal intensity for FITC-dextran, the mean green signal intensity was measured as the average of ten regions of interest (ROI) and normalized using the mean signal background intensity. The size and location of each ROI was consistent for all images. The mean signal intensity was measured and calculated using ImageJ version 1.52 k software (Bethesda, MD, USA) [68]. For quantification, the brain samples were weighted and homogenized. FITC intensity in the brain homogenates was determined using a fluorescent spectrometer with an excitation and emission spectrum of 485 nm and 520 nm, respectively. A standard curve was plotted using FITC-dextran and the results were expressed as µg/g brain tissue.

### 2.3. Evans Blue Assay

In addition to FITC-dextran, we used the Evans blue dye assay to determine the permeability of the BBB [69]. Briefly, rats received 4% of Evans blue (Sigma, St. Louis, MO, USA) through the intraperitoneal route (4 mL/kg). After 6 h post-administration, rats were perfused transcardially with PBS (Sigma, St. Louis, MO, USA) followed by the harvesting and weighing of brain tissues. Evans blue in brain tissue was extracted by homogenizing the samples in 0.1 mol/L PBS (pH 7.4), followed by protein precipitation using 60% trichloroacetic acid (Sigma, St. Louis, MO, USA). The samples were then vortexed and cooled. After 30 min, the samples were centrifuged and the concentration of Evans blue in the supernatant was determined at a wavelength of 610 nm using a spectrophotometer. A standard curve was plotted using Evans blue dye and the results were expressed as µg/g brain tissue. 

### 2.4. ZO-1 and iNOS Immunostaining 

For immunostaining, rat brains were harvested and fixed using 4% paraformaldehyde (Electron Microscopy Sciences, Hatfield, PA, USA) at 4 °C overnight. The samples were then cryopreserved by passing them through the sucrose gradient (5–30%) followed by embedding them in OCT compound media (Tissue-Tek, Sakura Finetek USA, Inc., Torrance, CA, USA) and allowing them to freeze at −20 °C. Sections of 10 µm were cut, blocked with 10% normal goat serum/5% BSA/PBST (0.3% Triton X-100), and stained overnight at 4 °C with the following primary antibodies: ZO-1 (1:100, Abcam, Waltham, MA, USA) and iNOS (1:100, Abcam, Waltham, MA, USA). After incubation, the samples were stained with Alexa Fluor 568 conjugated secondary antibody (ThermoFisher Scientific, Waltham, MA, USA) for 90 min at room temperature followed by mounting with the mounting medium containing DAPI (Vector Laboratories, Burlingame, CA, USA). The images were acquired using a confocal Zeiss Axiovert 700 microscope (Carl Zeiss Microimaging, LLC; Thornwood, New York, NY, USA). ImageJ version 1.52 k software (Bethesda, MD, USA) was used for processing and analyzing the images as well as for calculating the mean signal intensity as described for the FITC-Dextran assay [68].

### 2.5. Brain Glutathione (GSH) Levels

The levels of reduced glutathione (GSH) in the brain homogenates were determined as described in previous studies [70]. Briefly, the brain homogenate supernatant was added to trichloroacetic acid (10% *w*/*v*) (Sigma, St. Louis, MO, USA) in a 1:1 ratio, followed by centrifugation at 1000× *g* for 10 min at 4 °C. Next, 0.3 M disodium hydrogen phosphate (Sigma, St. Louis, MO, USA) was added to the supernatant followed by mixing with 0.001 M DTNB [5,5′-dithiobis (2-nitrobenzoic acid)] (Sigma, St. Louis, MO, USA). The absorbance of the samples was read at 412 nm. Using the reduced form of glutathione (Sigma, St. Louis, MO, USA), a standard curve was plotted, and the results were expressed as micromoles of reduced glutathione per mg of protein.

### 2.6. Determination of Brain Lipid Peroxidation by Measurement of Thiobarbituric acid Reactive Substances

To determine lipid peroxidation, brain thiobarbituric acid reactive substances (TBARS) levels were measured. The brain tissue supernatant was treated with 8.1% sodium dodecyl sulfate, 30% acetic acid (pH 3.5), and 0.8% thiobarbituric acid (all from Sigma, St. Louis, MO, USA) followed by incubation at 95 °C for 1h. The samples were then cooled, followed by the addition of n-butanol-pyridine mixture (15:1 *v*/*v*) (Sigma, St. Louis, MO, USA). The samples were then centrifuged, and the absorbance of the supernatant was determined using a spectrophotometer at a wavelength of 532 nm. A standard curve was prepared using 1,1,3,3-tetra methoxypropane (Sigma, St. Louis, MO, USA) and results were expressed as nM/mg protein. 

### 2.7. Nitrite Determination

The levels of NO were determined in the brain homogenates using the nitrite assay kit (Griess Reagent) (Sigma, St. Louis, MO, USA) as per the manufacturer’s instructions. A standard curve was plotted using sodium nitrite (Sigma, St. Louis, MO, USA) and the results were expressed as micromoles of reduced glutathione per mg of protein.

### 2.8. Statistical Analysis

All data was checked for the normal distribution through the Levene test using SPSS software version 28 (New York, NY, USA). If the samples were homogenous, the data analysis was performed using the student’s *t* test. In all other cases where samples were inhomogeneous, the statistical analysis of data was performed using the Mann Whitney test. *p* values < 0.05 were considered statistically significant.

## 3. Results

### 3.1. FITC-Dextran Assay

**Figure 1:**
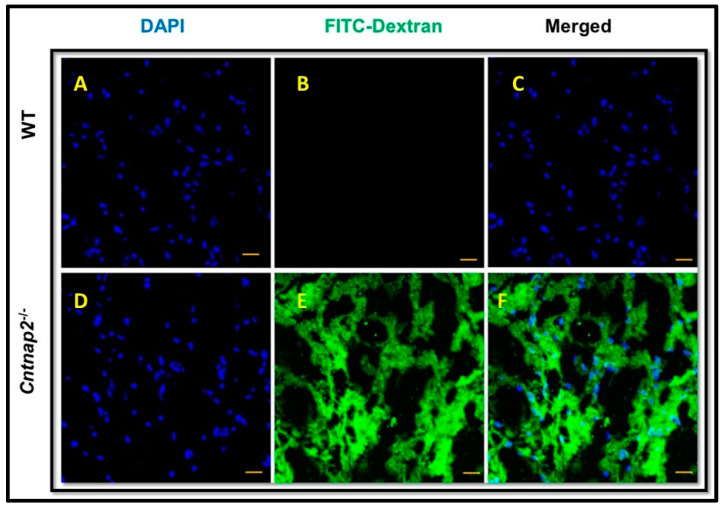
FITC-Dextran Assay: Representative photomicrographs of brain sections showing extravasation of the FITC-dextran as evident by green fluorescence staining. Intense green staining was observable in brain sections from *Cntnap2*^−*/*−^ rats (**D**–**F**) suggesting compromised BBB permeability compared to the littermate control WT group (**A**–**C**). Blue color indicates DAPI staining for cell nuclei. Scale Bars: 20 µM.

**Figure 2:**
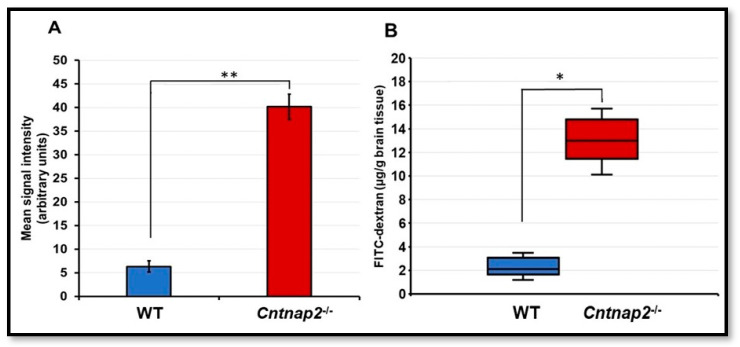
Quantification of FITC signal intensity: (**A**) Mean signal intensity for FITC staining was calculated using ImageJ software. (**B**) The FITC-dextran levels in brain homogenates were determined using fluorescent spectrophotometer. Data are expressed as mean values ± SD (N = 6 animals per group), * *p* < 0.01 or ** *p* < 0.001 *Cntnap2*^−*/*−^ compared to WT group.

To determine the integrity of the BBB, animals received intraperitoneal injection of FITC-dextran followed by harvesting of the brain samples after euthanasia. Confocal microscopy revealed a significantly high intensity of FITC-dextran in the brain slices of *Cntnap2*^−*/*−^ rats compared to the WT littermate control group (Figure 1A–F). There was a significant difference in the mean fluorescent intensity of the FITC-dextran signal in *Cntnap2*^−*/*−^ and WT rats (*p* < 0.001) (Figure 2A). Quantitation of FITC-dextran in the brain homogenates using a fluorescent spectrophotometer confirmed the confocal microscopy data showing significantly higher levels in *Cntnap2*^−*/*−^ rats suggesting compromised BBB (Figure 2B) (*p* < 0.01).

### 3.2. BBB Permeability Using Evans Blue Assay

**Figure 3:**
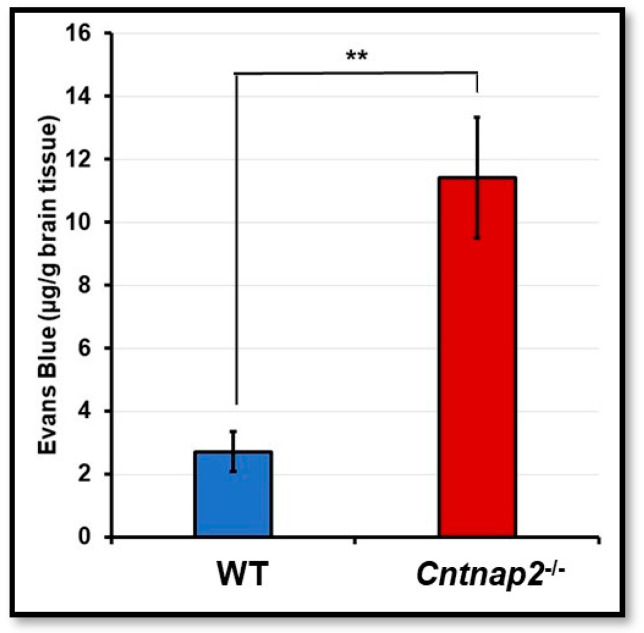
Evans blue assay: BBB integrity was determined using Evans blue assay. Quantification of Evans blue dye in brain homogenates revealed significantly high levels in *Cntnap2*^−*/*−^ rats compared to littermate control WT animals. Data are expressed as mean values ± SD (N = 6 animals per group). ** *p* < 0.01 *Cntnap2*^−*/*−^ compared to WT group.

To further confirm the results of the FITC-dextran assay, animals received Evans blue intraperitoneally to determine BBB permeability. The quantitative determination of Evans blue revealed significantly higher levels in the brain tissues of *Cntnap2*^−*/*−^ rats compared to WT littermate controls (*p* < 0.01) (Figure 3). The mean Evans blue levels were 11.41 µg/g brain tissue compared to 2.72 µg/g brain tissue in WT littermate controls. These results suggest that the BBB is compromised in *Cntnap2*^−*/*−^ rats.

### 3.3. ZO-1 Immunostaining

**Figure 4:**
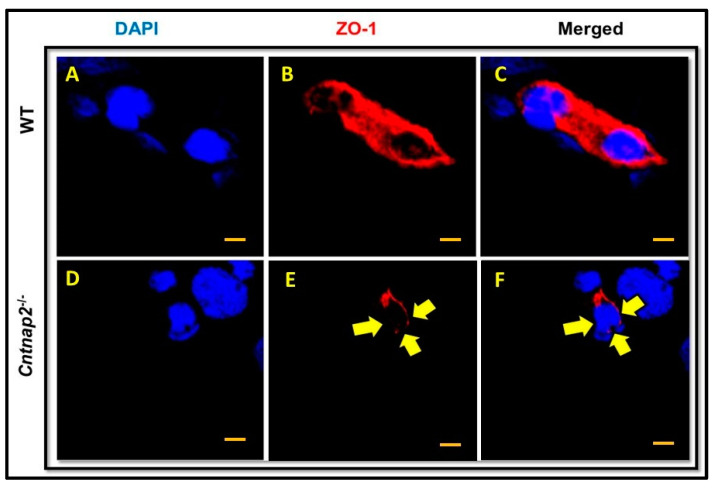
ZO-1 immunostaining: Representative photomicrographs of brain sections showing ZO-1 immunostaining. The brain sections from WT animals exhibited profound ZO-1 immunostaining with a pattern around the cell margins (**A**–**C**). On the contrary, there were gaps and disruptions in ZO-1 immunostaining in brain sections from *Cntnap2*^−*/*−^ rats (**D**–**F**) (indicated by yellow arrows). Scale Bars: 20 µM.

**Figure 5:**
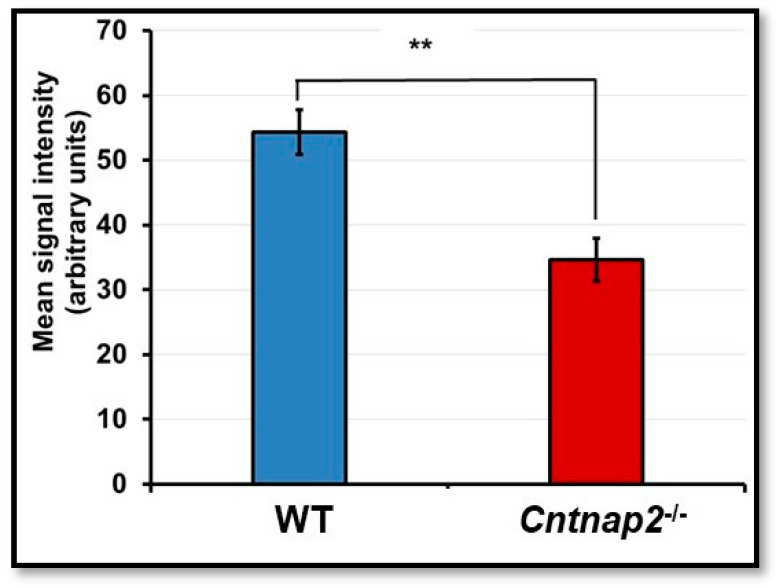
Mean signal intensity for ZO-1 immunostaining: ImageJ software was used to determine mean signal intensity for ZO-1. ** *p* < 0.01 *Cntnap2*^−*/*−^ compared to WT group.

ZO-1 is one of the major tight junction proteins and is an integral part of the BBB. The changes in levels of ZO-1 have been associated with BBB damage and increased permeability. Therefore, we determined ZO-1 protein expression in brain sections of *Cntnap2*^−*/*−^ and WT rats using immunostaining. Brain sections from WT rats showed intense ZO-1 staining with a continuous pattern along the cell margin (Figure 4A–C). On the other hand, there were gaps and disruptions in ZO-1 staining in brain sections from *Cntnap2*^−*/*−^ rats (Figure 4D–F). The mean fluorescent intensity data confirmed that ZO-1 expression was significantly reduced in the brain tissues of *Cntnap2*^−*/*−^ rats compared to WT animals (*p* < 0.01) (Figure 5). The mean signal intensity for ZO-1 immunostaining was 34.66 arbitrary units in *Cntnap2*^−*/*−^ animals compared to 54.33 arbitrary units in WT rats.

### 3.4. Oxidative Stress

**Figure 6:**
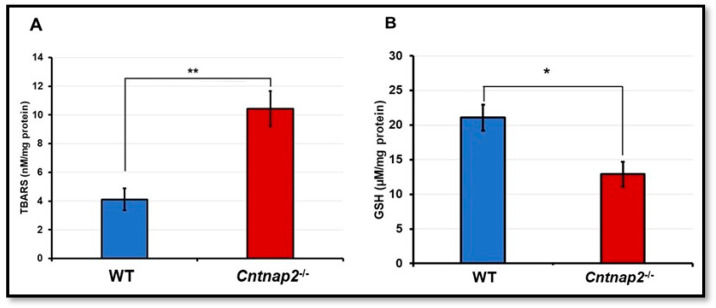
Oxidative stress determination: The lipid peroxidation (**A**) and levels of glutathione (**B**) were determined in brain homogenates of WT and *Cntnap2*^−*/*−^ rats as markers of oxidative stress. Data are expressed as mean values ± SD (N = 6 animals per group). * *p* < 0.01 or ** *p* < 0.001 *Cntnap2*^−*/*−^ compared to WT group.

The brain lipid peroxidation and glutathione levels were used as surrogate markers of oxidative stress. To determine lipid peroxidation, brain thiobarbituric acid reactive substances (TBARS) levels were measured. TBARS levels were significantly higher in brain homogenates of *Cntnap2*^−*/*−^ rats compared to the WT littermate controls (*p* < 0.001) (Figure 6A). On par with these findings, there was a significant difference in the reduced glutathione (GSH) content in the brain homogenates of *Cntnap2*^−*/*−^ and WT rats. The reduced glutathione level in *Cntnap2*^−*/*−^ rats was 12.9 µM/mg protein where it was 21.08 µM/mg protein in WT littermate controls (*p* < 0.01) (Figure 6B). 

### 3.5. Nitrosative Stress in Cntnap2^−/−^ Rats

**Figure 7:**
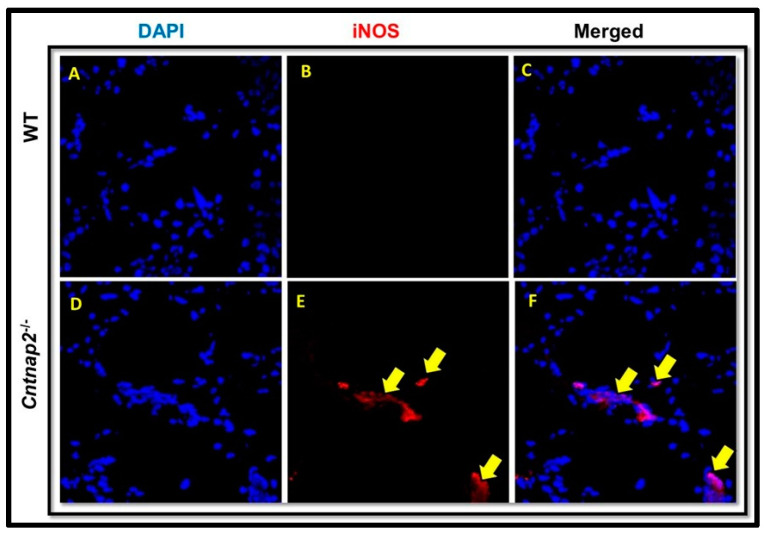
iNOS immunostaining: Representative photomicrographs of brain sections showing iNOS immunostaining. The brain sections from WT rats showed undetectable levels of iNOS immunostaining (**A**–**C**). On the other hand, intense iNOS immunostaining was evident in brain sections from *Cntnap2*^−*/*−^ rats (**D**–**F**) (indicated by yellow arrows). Red color: iNOS immunostaining; Blue color: DAPI staining for cell nuclei. Scale Bars: 20 µM.

**Figure 8:**
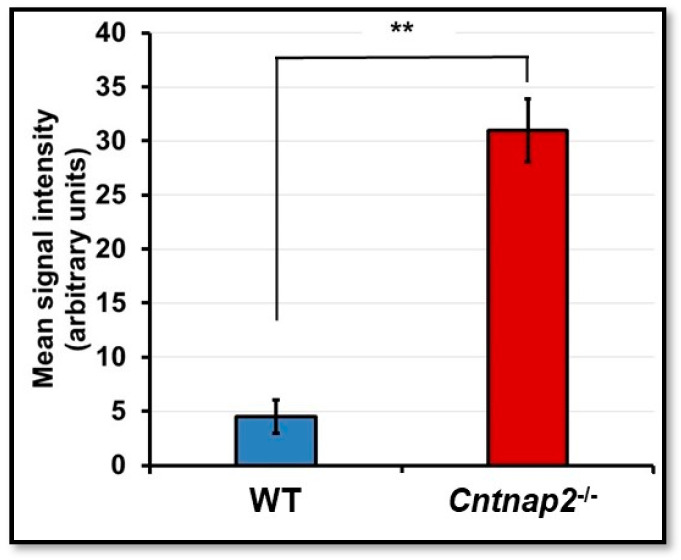
Mean signal intensity for iNOS immunostaining: ImageJ software was used to determine mean signal intensity for iNOS. ** *p* < 0.001 *Cntnap2*^−*/*−^ compared to WT group.

To determine nitrosative stress, iNOS immunostaining was performed on brain cryosections. The brain sections from WT animals showed undetectable staining (Figure 7A–C), whereas intense iNOS staining was evident in the brain sections from *Cntnap2*^−*/*−^ rats (Figure 7D–F). iNOS immunostaining was seen in a few cells and not at all the cells as very high levels of nitrosative stress will become lethal and may lead to mortality in animals. Mean signal intensity for iNOS immunostaining was 31 arbitrary units in brain sections of *Cntnap2*^−*/*−^ rats compared to 4.5 arbitrary units in WT animals (Figure 8).

**Figure 9:**
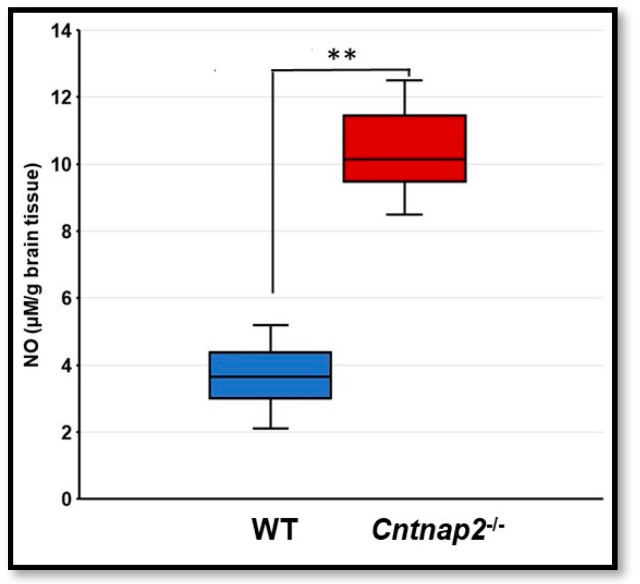
Nitric oxide (NO) determination: The levels of NO were determined in brain homogenates using Griess reagent. Data are expressed as mean values ± SD (N = 6 animals per group). ** *p* < 0.001 *Cntnap2*^−*/*−^ compared to WT group.

For quantitation, nitrite levels were determined in brain homogenates using Griess reagent. There was a significant increase in nitrite levels in the brain homogenates from *Cntnap2*^−*/*−^ rats compared to WT littermate controls (*p* < 0.001) (Figure 9). The mean NO levels in the brain homogenates of WT and *Cntnap2*^−*/*−^ rats were 3.66 and 10.36 µM/g brain tissue, respectively.

## 4. Discussion

The blood-brain barrier (BBB) is a selective and tightly regulated barrier that separates the vascular compartment from the central nervous system [71,72,73]. The main function of the BBB is to protect the brain from pathogens as well as maintaining homeostasis by regulating the entry of solutes and other foreign substances into the brain [74]. A number of neurological disorders have been associated with the disruption of the BBB, such as Alzheimer’s disease, and ASD [53,56,75,76,77,78]. It is interesting to note that BBB disruption can lead to different neurological disorders, depending on the inflammatory molecules and pathological proteins present in the milieu. For example, BBB disruption allows tau proteins to enter the brain which has been implicated in the pathology of Alzheimer’s disease [79,80]. On the other hand, enhanced BBB permeability leads to the entry of inflammatory molecules (such as TNF-α) and free radicals such as nitric oxide which may determine predisposition to ASD [81]. To gain a better understanding of BBB disruption and leakage, various tracers are currently used. One of the most popular tracers for assessing the permeability of the BBB is fluorescein isothiocyanate (FITC) labeled dextran [67,82,83]. Due to the fluorescein moiety that can be measured even in low concentrations, FITC-dextran serves as a sensitive and reliable marker to determine BBB permeability [67]. In this study, we observed that there was a strong FITC signal in the brains of *Cntnap2*^−*/*−^ rats compared to the WT littermate control group, suggesting BBB disruption. Quantitation of FITC in brain tissue homogenates confirmed these findings showing a significant amount in the brains of *Cntnap2*^−*/*−^ rats than the control group and thus indicating a compromised BBB. Another well-accepted technique to determine BBB integrity is penetration of Evans blue dye into the brain [84,85,86,87,88,89]. On par with our FITC permeability data, we observed significantly high amounts of Evans blue dye in the brain homogenates of *Cntnap2*^−*/*−^ rats, whereas very low levels were detectable in the brain tissues of the control group suggesting impaired BBB permeability. These findings are in agreement with previously published studies. The increased inflammatory milieu prevalent in ASD has been implicated in BBB disruption [61,81,90]. In addition, decreased expression of adhesion molecules that modulate the permeability and signaling at the blood–brain barrier as well as leukocyte infiltration into the CNS, such as platelet endothelial adhesion molecule-1 (PECAM-1), intercellular adhesion molecule-1 (ICAM-1), vascular adhesion molecule-1 (VCAM-1), P-selectin, and L-selectin can further affect the integrity of the BBB [91,92,93]. This BBB disruption has been demonstrated in preclinical animal models of ASD [94]. Furthermore, an altered expression of genes associated with BBB integrity along with increased neuroinflammation has been observed in postmortem brain samples from human ASD subjects [81]. The increased BBB permeability observed in this study along with previous studies suggests that *Cntnap2*^−*/*−^ rats have a high construct and face validity and may be useful to better understand the mechanisms involved in ASD. 

Tight junction proteins are an integral component of the BBB [74,95]. Endothelial tight junction and adherens junction proteins contribute to the physical barrier of the BBB [96]. Tight junctions between brain microvascular endothelial cells are the first barrier to maintain cerebral homeostasis. These tight junctions are composed of occludins, claudins and zonula occludens 1,2, and 3 (ZO-1, ZO-2, and ZO-3) [96]. ZO-1 is one of the major tight junction proteins that facilitates in maintaining the BBB integrity. ZO-1 can help in predicting the healthy and pathological state of the BBB, making it a valuable marker of the endothelial barrier [97]. Decreased expression and disarrangement of ZO-1 has been associated with an increase in BBB permeability [98]. In the present study, we observed decreased expression and disruption in ZO-1 immunostaining in brain sections from *Cntnap2*^−*/*−^ rats. This decreased expression of ZO-1 may have contributed to the increase in BBB permeability observed in *Cntnap2*^−*/*−^ rats.

The term oxidative stress refers to the imbalance between the production of reactive oxygen species (ROS) and the antioxidant capacity of cells [99]. The antioxidant defense mechanisms neutralize the excess production of ROS providing protection against oxidative stress. Glutathione, catalase, and superoxide dismutase (SOD) are potent antioxidant defense mechanisms. ROS are the product of the cellular mechanism, however, an increase in free radical activity causes DNA and protein damage, as well as lipid peroxidation, which can lead to cell damage and cell death. Studies have demonstrated the pertinent role of oxidative stress in the pathophysiology of neurological disorders such as ASD [100]. In fact, associations between markers of increased oxidative stress and the severity of ASD have been observed [100,101]. Increased levels of lipid peroxidation in blood plasma have been seen among children with ASD when compared to their neurotypical siblings [34,35]. Specifically, markers of lipid peroxidation such as TBARS and aminoglycerophospholipids (AGPs) have been seen in significantly increased levels in the blood plasma among children with ASD compared to neurotypical control group [35,101,102]. On par with these findings, we observed significantly high levels of TBARS in the brain homogenates of *Cntnap2*^−*/*−^ rats compared to the WT littermate controls, suggesting significant lipid peroxidation.

Glutathione is vital in protecting cells from oxidant damage [103], however, glutathione redox imbalance is commonly seen in individuals with ASD. In ASD, decreased concentrations of reduced glutathione (GSH) and greater levels of oxidized glutathione (GSSG) have been observed, as well as a decreased GSH/GSSG redox ratio [101,104,105,106]. Additionally, decreased glutathione levels within red blood cells (RBCs) have been associated with ASD severity [107,108]. In the present study, we observed a significant decrease in GSH levels in the brain homogenates of *Cntnap2*^−*/*−^ rats compared to the control group, suggesting oxidative stress.

Nitric oxide (NO) is an important cellular signaling molecule generated by the nitric oxide synthase (NOS) enzyme through oxidation of L-arginine to L-citrulline. Of the three NOS isoforms, nNOS and eNOS are constitutively expressed in the brain while iNOS is expressed under pathological conditions. NO plays a vital role in the neurodevelopmental process in the CNS. However, excessive production of iNOS induces nitrosative stress and has been implicated in the pathophysiology of various neuropsychiatric disorders such as sepsis, multiple sclerosis and ASD [109,110,111,112]. In a study with the *SHANK3* mutated mouse model showed that, involvement of NO-related molecular changes in the brain might affect the development of ASD [113]. Elevated levels of NO have been found in the blood plasma, saliva, urine, and cerebrospinal fluid of individuals with ASD [50,51,114]. Our results are in agreement with these findings showing high levels of iNOS and NO suggesting nitrosative stress in *Cntnap2*^−*/*−^ rats.

One of the limitations of our study is that we used rats, which have constitutive deletion of *Cntnap2*. Further studies using conditional knockout rats with deletion of *Cntnap2* only in the brain can shed more light on the role of *Cntnap2* signaling in the brain and predisposition to ASD.

Although *CNTNAP2* has been proposed to be implicated in ASD [14,15,16,17,18,19,20,21,22,23], recent studies with large datasets suggest a neutral or less penetrant role for genetic variants of this gene in ASD [27,28,29]. 

Homozygous *CNTNAP2* deletions have been shown to cause a monogenic disease called Pitt-Hopkins-Like Syndrome 1 (PTHLS1) [115,116,117]. PTHLS1 is a rare Mendelian condition characterized by severe intellectual disability, behavioral abnormalities, psychomotor delay along with other symptoms of facial dysmorphism, stereotypic movements, breathing difficulties, and seizures [116]. Some of these patients also present autism-like behaviors. Recapitulating PTHLS1 in rodent models can provide a great opportunity to understand this mendelian condition and some clinical manifestations resembling ASD.

Despite conflicting results of *CNTNAP2* variants in predisposition to ASD, it is generally accepted that *CNTNAP2* signaling is important for normal functioning of the brain by influencing synaptic plasticity and neurotransmission [24,25,26]. A study showed that *CNTNAP2* heterozygous variants may contribute to the pathophysiology of ASD [118]. Using cortical neuronal cultures from wild-type, *Cntnap2^+/−^* and *Cntnap2*^−*/*−^ embryos at E14.5, it was observed that loss of one *Cntnap2* allele is sufficient to elicit axonal growth alteration, which may hamper neurodevelopment and neurotransmission. The authors of this study suggested that these findings may recapitulate the clinical situations which may be relevant for *CNTNAP2* heterozygosity in individuals with ASD [118]. 

In summary, our results suggest increased BBB permeability and oxidative stress in *Cntnap2*^−*/*−^ rats similar to the findings observed in individuals with ASD. The ASD-like phenotype as reported in previous studies correlated well with our molecular/histological alterations observed in this study. The *Cntnap2*^−*/*−^ rat model may be explored to decipher the role of BBB permeability and oxidative stress in the predisposition to ASD. The behavior deficits and auditory dysfunction observed in previous studies [62,63,64,65,66] as well as the oxidative stress and BBB permeability observed in this study make *Cntnap2*^−*/*−^ rats an appropriate model to determine the efficacy of novel effective preventive and therapeutic strategies for ASD. The availability of novel therapeutic modalities for ASD will lead to improved quality of life of affected individuals and their families.

## Data Availability

Not applicable.
